# Colon cancer survival in the elderly without curative surgery

**DOI:** 10.1308/rcsann.2023.0059

**Published:** 2024-02-26

**Authors:** J Franklyn, A Poole, I Lindsey

**Affiliations:** Oxford University Hospitals NHS Foundation Trust, UK

**Keywords:** Colon cancer, Cancer

## Abstract

**Introduction:**

The aim of this study was to chart the natural history of elderly patients with colon cancer who are managed nonoperatively, with the primary outcome being life expectancy from diagnosis to death.

**Methods:**

This was a retrospective analysis of patients aged 80 years and above diagnosed with colon cancer in a tertiary care referral hospital in England between 1 January 2012 and 31 December 2017.

**Results:**

Thirty-two patients were diagnosed with non-metastatic colon cancer and managed non-operatively. The median age of patients in this study was 86 years. The group had a median Charlson Comorbidity Index of 7 (range 6–12) and the median frailty score was 6 (range 3–8). Progression to metastatic disease was identified in two patients; two further patients showed locoregional progression of cancer and therefore required palliative surgical intervention. Survival of these patients ranged from 105 to 1,782 days with a median life expectancy of 586 days. Place of death was identified in 15/31 patients: 4 (27%) died in hospital, 12 (38%) died at home and 15 (47%) died in a nursing or residential home; data were missing for 1 patient (3%).

**Conclusions:**

Nonoperative management of elderly patients with colon cancer yields reasonable life expectancy and a low risk of life-threatening local complications.

## Introduction

There are more than 1.6 million people in England over the age of 85 years, with this figure likely to double by 2043, and more than 40% of colorectal cancer patients are aged 75 years and over.^1^ Despite advances in anaesthesia and perioperative care, surgery in frail, older patients with non-metastatic operable colon cancer can be a daunting task. At the same time, the natural history of patients managed conservatively is unknown because the role of nonoperative management of colon cancer in elderly patients has not been studied as a separate entity in England.^2,3^ Unlike rectal cancer, there is a lack of non-surgical treatment options for local disease control. Therefore, the decision to offer nonoperative management of colon cancer must be weighed carefully against any potential adverse outcomes.

The aim of this study was to chart the natural history of elderly patients with non-metastatic, non-obstructive colon cancer who are managed nonoperatively.

## Methods

This was a retrospective analysis of patients aged 75 years and above diagnosed with colon cancer in a tertiary care referral hospital in England between 1 January 2012 and 31 December 2017.

Patients with clear radiological or pathological colon cancer diagnosis who were not for surgical management or for further investigations were included. Patients with metastatic cancer or those who underwent palliative resection were excluded from the study.

**Figure 1:**
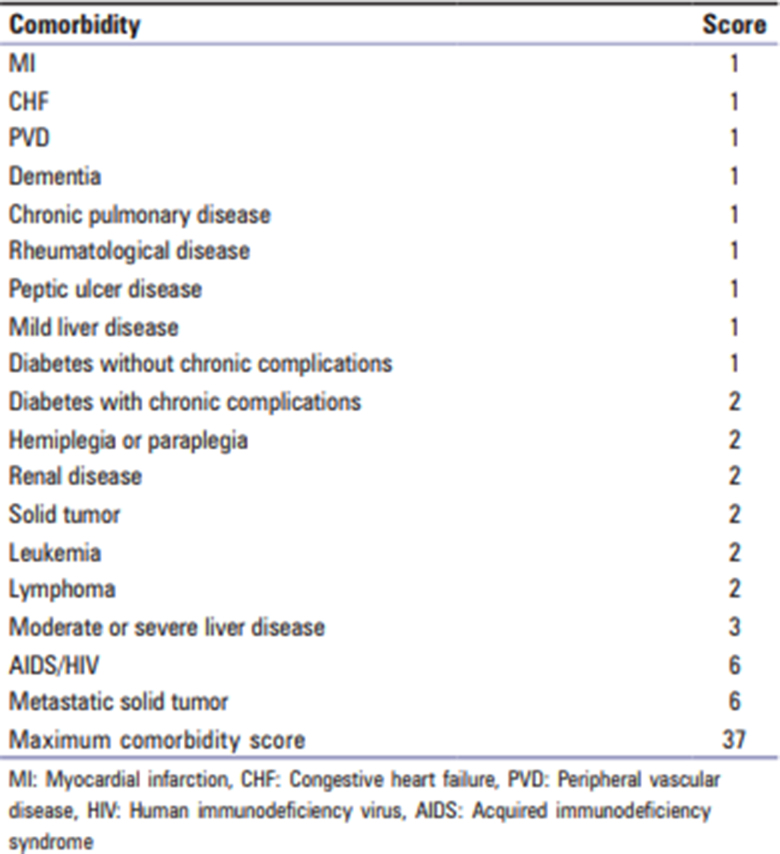
Charlson Comorbidity Index

**Figure 2:**
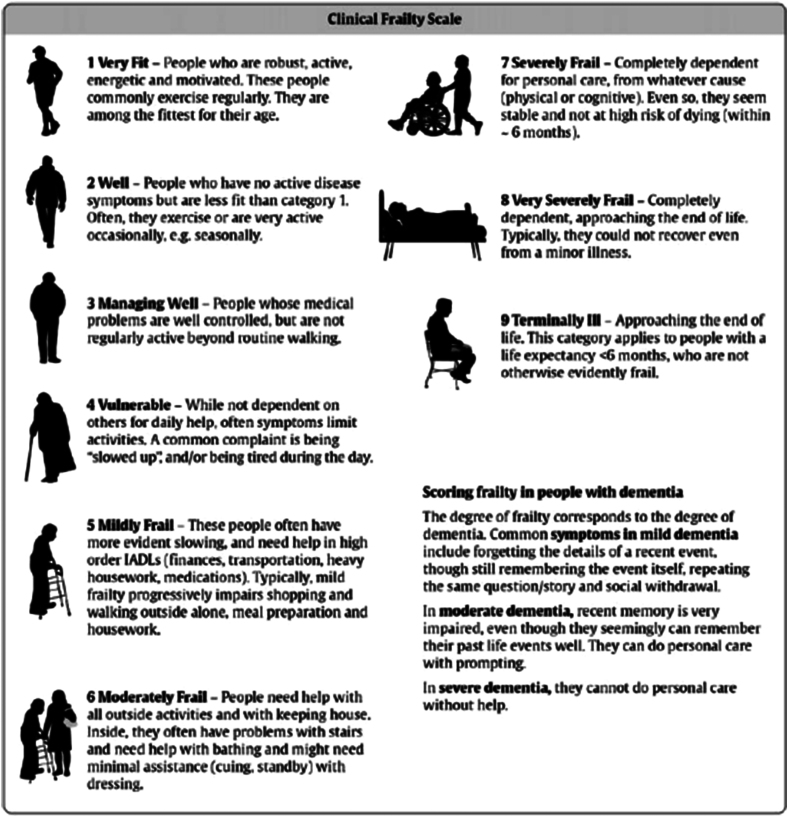
Clinical frailty assessment

Patients who met the inclusion criteria were identified from a prospectively maintained database. Comorbid illness status (Charlson Comorbidity Index [CCI] and frailty score) (Figures 1 and 2) and details of survival were obtained from the hospital database.^4,5^ In addition, a cardiopulmonary exercise test (CPET) was utilised in patients with borderline operability.^6^ We used the American Joint Committee on Cancer system 7th edition to stage cancers and all patients were discussed in a colorectal multidisciplinary meeting (MDT) before deciding on their management plans. The study was reported in accordance with STROBE guidelines.^7^

The primary outcome measured was to quantify the natural chronology of patients from diagnosis when nonoperative management was considered in elderly patients with colon cancer.

A secondary outcome was to quantify the time of progression to metastatic disease or intervention.

## Results

During the study period, 32 patients fulfilled the inclusion criteria. The median age of patients in this study was 86 years (range 76–98 years). The male-to-female ratio, distribution of cancer in colon and stage at diagnosis are given in Table 1.

**Table 1:** Demographics of elderly patients with colon cancer managed nonoperatively

	Nonoperative management of colon cancer * n* (%)
Sex	
Male	11 (34.4)
Female	21 (65.6)
Distribution	
Right colon	18 (56.3)
Transverse colon	2 (6.3)
Left colon	1 (3.1)
Sigmoid	8 (25.0)
Rectosigmoid	1 (3.1)
Unspecified	2 (6.3)
Stage at diagnosis	
T1	0 (0)
T2 N0 M0	2 (6.3)
T2 N1 M0	3 (9.4)
T3 N0 M0	4 (12.5)
T3 N0 Mx	1 (3.1)
T3 N1 M0	3 (9.4)
T3 N2 M0	2 (6.3)
T4 N0 M0	2 (6.3)
T4 N1 M0	2 (6.3)
T4 N1 Mx	1 (3.1)
T4 N2 M0	5 (15.6)
Not staged	7 (21.9)

The group had a median CCI of 7 (range 6–12) and a median frailty score of 6 (range 3–8). CPET was done in six patients. Using multiple metrics and clinical assessment, a decision to provide nonoperative treatment was made by the consultant surgeon in 29/32 patients.

During follow-up, progression to metastatic disease was identified in two patients and two further patients showed locoregional progression of cancer. The various nonoperative treatments that patients received are listed in Table 2.

**Table 2:** Management of local complications following conservative treatment of colon cancer

	Palliative treatment offered *n* (%)
Colonoscopic stenting	2 (6.3)
Palliative stoma	1 (3.1)
Palliative radiotherapy	0
Palliative chemotherapy	0

The life expectancy of patients managed conservatively ranged from 105 to 1,782 days with a median life expectancy of 586 days.

**Figure 3:**
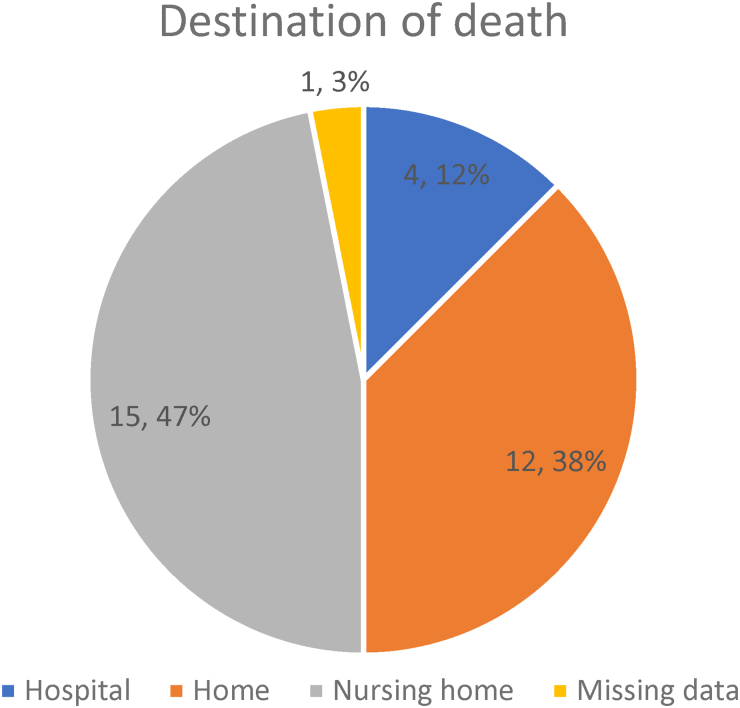
Destination of death when colon cancer is managed nonoperatively

Figure 3 outlines the common places where patients at the end of life die, which reveals that the majority of patients managed conservatively die at home or in a care facility rather than in a hospital.

## Discussion

The 85+ age group is the fastest growing cohort in England, and poised to double by 2043 and treble to 5.1 million people in the latter half of the 22nd century.^8^ Despite four in ten new colorectal cancers being diagnosed in those aged above 75, these patients have been significantly under-represented in clinical studies.^9^ To compound this knowledge gap, most retrospective case series studying this age group have combined colon and rectal cancers.^10^ The investigational and management approach to both these cancers are different. This makes it all the more difficult to inform a concerned relative or patient of the prognosis if nonoperative management is chosen for colonic malignancy.

The results of this retrospective study show that the median survival of patients diagnosed with non-obstructed, non-metastatic colon cancer is over a year and a half (range 105–1,782 days). This is similar to results from other studies in England.^11^ However, most studies have combined colorectal cancers or focused solely on rectal cancer.^10^ The average life expectancy of an 85-year-old in England is 91 years for men and 92 years for women.^12,13^ Interestingly, survival for patients in this study ranged from a few days to more than 5 years. The ability to scientifically predict the person who is likely to survive 3–5 years without a resectional surgery should be the focus of future research. At present, this is largely subjective and the tools to gauge fitness are flawed. The CPET, frailty assessment and the surgeon’s clinical assessment in a busy clinic are crude metrics to define and decide who benefits from an operation. The presence of a geriatrician or nurse specialist who can add social and background medical context may be useful in the management of colorectal cancer of the elderly, more so in colon cancer.

A literature search revealed no articles that focused solely on the conservative management of colon cancer, and it is safe to state that colon cancer does not receive the same research impetus and detailed decision-making that rectal cancer receives. In many hospitals, colon MDTs are held separately and most decisions go through automatically when compared with the more nuanced detailing of the management of rectal cancer. This is usually justified because the approach to rectal cancer is no doubt more complex. However, in the context of colon cancers in the elderly, there are fewer curative treatment options in the clinician’s armament (such as radiation therapy). By contrast, stenting of colon cancers is usually more successful than in rectal cancers. The recent role of neoadjuvant therapy resulting in tumour shrinkage opens up newer models that need to be explored in this age group as well.^14^ It is therefore important that elderly colon cancer patients are treated holistically in a multidisciplinary manner, rather than being recipients of a binary decision algorithm, wherein the surgeon is asking themselves whether ‘to operate or not to operate’.

### Study limitations

Inherent to the study design, a retrospective case series is prone to information and selection bias. In addition, in the elderly population, quality of life is important; unfortunately, these data are not available and we have assessed cancer outcomes numerically as years lived following diagnosis. This is a single-centre case series, thereby limiting generalisability.

Despite these limitations, to our knowledge, this is the largest case series of colon cancers managed nonoperatively. We are optimistic that the results of this paper will provide baseline data, identify lacunae and encourage further research in this evolving healthcare topic.

## Conclusion

In appropriately selected patients with non-metastatic, non-obstructed colon cancer, conservative management yields reasonable life expectancy and a low risk of life-threatening local complications. However, current tools to decide and deny treatment are subjective and we conclude that newer methods are required to carefully select patients with colon cancer for nonoperative management.
